# Identified obstacles and prerequisites in telenurses’ work environment – a modified Delphi study

**DOI:** 10.1186/s12913-017-2296-y

**Published:** 2017-05-18

**Authors:** Annica Bjorkman, Maria Engstrom, Annakarin Olsson, Anna Carin Wahlberg

**Affiliations:** 1 Faculty of Health and Occupational Studies, Caring Science University of Gavle, Gavle, Sweden; 2 Department of Public Health and Caring Sciences, Uppsala University, Health Services Research, Uppsala, Sweden; 3 Nursing Department, Medicine and Health College, Lishui University, Lishui, People’s Republic of China; 4 Department of Neurobiology, Care Sciences and Society, Karolinska Institutet, Stockholm, Sweden

**Keywords:** Nursing, Telenursing, Work environment, Delphi-study

## Abstract

**Background:**

Telenursing is an expanding part of healthcare, staffed with registered nurses whose work environment is typical of a call centre. Work-related stress has been shown to be a major problem in nurses’ work environments and of importance to the outcome of care, patient safety, nurse job satisfaction and burnout. Today, however, we have a limited understanding of and knowledge about the work environment for telenurses. The aim of the present study is to explore and reach consensus on perceived important obstacles and prerequisites in telenurses’ work environment.

**Methods:**

A modified Delphi design, using qualitative as well as quantitative data sequentially through three phases, was taken. Data were initially collected via semi-structured interviews (Phase I) and later using a web survey (Phase II-III) between March 2015 and March 2016.

**Results:**

The findings present a consensus view of telenurses’ experiences of important obstacles and prerequisites in their work environment. Central to the findings are the aspects of telenurses having a demanding work, cognitive fatigue and having no opportunity for recovery during the work shift was ranked as important obstacles. Highly ranked prerequisites for managing were being able to focus on one caller at a time, working in a calm and pleasant environment and having technical support 24/7.

**Conclusions:**

Managers need to enable telenurses to experience control in their work, provided with possibilities to control their work and to recover during work; shortening work time could improve their work environment. Limited possibilities to perform work might contribute to feelings of stress and inability to perform work.

## Background

Telephone advice nursing (henceforth *telenursing*) is an expanding part of healthcare in many western countries, including the UK, the US, Canada, Australia, Denmark and the Netherlands [1, 2]. Telenursing services are organized in call centres and intended to steer patient flows so as to avoid unnecessary patient visits and ease the strain on limited healthcare resources. Telenursing services are staffed with registered nurses (RNs, henceforth *telenurses*). Their work environment is typical of a call centre, in that they sit in front of a computer wearing headsets and handling incoming calls [2, 3]. During a short period of time, telenurses make an assessment of the care-seekers need for care, independently triage the need for care, give individualized care (e.g., give self-care advice, information and support), and refer the care-seeker to the appropriate healthcare provider, all using only communicative strategies [1, 2]. Telenurses do not only receive calls from their own county council/region but over the entire country due to collaboration among call-centre sites. If a call-centre have long telephone ques, calls will be forwarded to a call-centre with the shortest que. Hence, the work also demands that telenurses be well versed in healthcare organization [4, 5]. Placing the caring nature of nursing within a call centre context is not uncomplicated; previous studies [6, 7] have shown how telenurses are exposed to conflicting demands and how employees call for efficiency as well as unequivocal and evidence-based advice. These conflicting demands are likely to lead to experiences of stress. Work-related stress has been shown to be a major problem in nurses’ work environment in other fields of nursing [8–10]. Work environment has also shown to be of importance to the outcome of care, patient safety [11], nurse job satisfaction and burnout [11, 12] within other fields of nursing. Today, however, there is little understanding of and knowledge about the work environment for telenurses. The present study aims to explore and reach consensus on perceived important obstacles and prerequisites and in telenurses’ work environment.

In Sweden, the national healthcare service Swedish Healthcare Direct (SHD) was introduced during 2003 and is today one of Sweden’s largest healthcare providers, with approximately 5.5 million citizens contacts during 2015. SHD has 23 different call centres spread throughout the country. The Swedish service is quite similar to the UK service NHS Direct, the difference being that Sweden does not have call handlers who first triage callers’ need for care and forward the appropriate calls to the telenurses. SHD is staffed by approximately 1100 telenurses [3] with the assistance of a computerized decision support system (CDSS). The CDSS used at SHD, as well as at NHS Direct, can be entered either based on symptoms or diagnosis, and it covers various symptoms and medical conditions among children, adolescents, adults and older people [4].

The work environment at SHD (and NHS Direct) is typical of a traditional call centre, in that all patient contacts are made via telephone without any possibility to have physical contact with the care seeker. At SHD, all calls are recorded and call time, number of answered call per hour, level of referral and queue time are registered, monitored and evaluated [3, 13]. Telenurses employed at SHD have described how they perceive a conflict between nursing care, where every patient should be treated as an individual, and managers’ demands for efficiency, with short queue times and short calls [7, 14]. According the quality goals of SHD, calls should be answered within 3 min, and during periods of increased illness (e.g., influenza season), long telephone queues are common [14]. Previous studies have shown that telenurses feel long queue times lead to dissatisfaction and threats from callers [7, 14]. The telenurses cannot influence the number of calls to SHD; all calls are forwarded to the telenurses and placed in the telephone queue. Telenurses working at SHD are constantly reminded of the actual length of the telephone queue via their computer [15].

Karasek and Theorells [16] model describes how the relationship between demands and control in work determine if the work leads to positive or negative stress. Work situations with high psychological demands and low decision latitude, such as nurses and telephone operators is at greater risk of developing work related illness compared to work situations with high psychological demands and high decision latitude such as engineers and physicians. The demand – and control model describes how the effects of psychological demands on employees co-variates with the level of control, hence acting space and decision latitude. The effects of psychological demands also co-variates with the level of support offered from the organization to the employees. According to the model, the worst work situation is iso-tensed situations whereas the employee is exposed to high demands, with low levels of support and low possibility to influence. According to the literature, there is a consensus that iso-tensed work is a risk factor for the development of cardiovascular diseases [16].

Telenursing is a complex kind of care, telenurses create a picture of the caller’s condition using communicative strategies only. In addition to asking the right questions, the telenurses must use non-verbal communicative strategies to verify or exclude serious symptoms [17]. It is also essential that telenurses, though communication, enable trust and a caring relationship with the caller [18]. If the telenurse misjudges the caller’s need for care, the consequences may be fatal and lead to malpractice claims against the individual telenurses. To enable adequate and safe nursing care, nurses need feedback on their work. This is accomplished daily within traditional care, where continuity of patient care is more common. Within telenursing, the opportunity for feedback is lacking, because telenurses are never given the possibility to follow-up on callers.

Work-related stress is a major problem within nurses’ work environment and leads to physical illness [19] as well as decreased job satisfaction. Stress can also result in impaired patient satisfaction with care provision [20], insufficient quality of care [21], increased patient mortality [20], and increased risk of medical errors. In a study investigating medical errors within SHD, the results showed that work stress and unclear work descriptions (lack of information) were identified as contributing factors. Secondary findings from previous studies conducted within the research team have indicated deficiencies in these factors for telenurses working within SHD [14, 15, 22–24]. The telenurses experience conflicts within their work, stressful situations and problems referring the caller to the appropriate healthcare provider [23]. Access to essential structural conditions has shown fewer stress symptoms, higher job satisfaction [25] and improved work effectiveness [26–28]. Today, there is little understanding of and knowledge about the work environment, obstacles and important prerequisites in telenursing.

The concept of work environment is here understood as involving several parts, the physical work environment, the psychological and the social work environment, the latter commonly described as the psychosocial work environment. Physical work environment relates to aspects such as light, surrounding sound and tool needed to perform work such as computers, desks and chairs. Psychological work environment relates to work load and work related stress. Social work environment relates to aspects of cooperation between colleagues, social relations at work and support within the work group. Psychosocial work environment relates to aspects such as stress, control, demand, leadership, communication, influence/power, social support and balance between work and private life [16, 28].

The present study is a part of a research project focusing on telenurses’ work environment. Because work environment has been shown to be important to the outcome of care [11], the present study aims at exploring and reaching consensus on perceived important obstacles and prerequisites in telenurses’ work environment, hence at identifying potential deficits and thereby enabling interventions that promote a healthy and productive work environment.

## Methods

### Aim

The aim of the study were to explore and reach consensus on perceived important obstacles and prerequisites in telenurses’ work environment.

### Design and setting

**Fig. 1:**
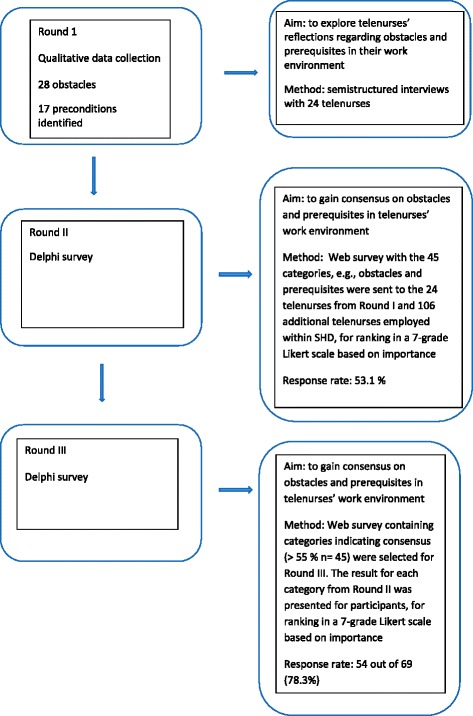
A description of methods, participants and sample size

A modified Delphi design, using qualitative as well as quantitative data sequentially through three phases, was taken. A description of the data collection method and sample size is presented in Fig. 1. The Delphi design was chosen because it is widely used within healthcare research [29, 30] to explore and gain consensus within a group of participants who have experience of the topic of interest to the researchers [29]. The SHD service is provided via 23 call centers, located throughout the country, linked via a network. The study included participants from six call centers located in different parts of Sweden and the centers varied on the dimensions, e.g., geographical location, large (more than 1.4 inhabitants) vs. small (22.000 inhabitants) city, northern vs. southern Sweden.

### Participants

For Round I, the inclusion criterion was having worked at the present workplace for at least half a year at the time of the interview. Twenty-four telephone advice nurses, all female, between 33 and 67 years of age (mean 55.4 SD 1.7) with 2.5 to 35 years’ (mean 6.2 SD 0.9) experience of telephone advice nursing participated in the interviews. For Round II and III, an additional 106 telenurses employed within SHD were invited to participate in the web survey. The participants in Round II and III varied in age from 33 to 73 years (mean 56.5 SD 0.9) with 7 months to 33 years’ (mean 6.9 SD 0.5) experience of telephone advice nursing. Two of the participating telenurses were male. The participating telenurses worked for pubic non-profit as well as private for-profit provider organizations, distributed throughout the country.

### Data collection

The data collection was conducted between March 2015 and March 2016 in three rounds, and the results from each round of the Delphi are presented in the results section. An overview of the methods and participants for each round of the study is presented in Fig. 1. Data were initially collected via semi-structured interviews (Round I) (n = 24) using an interview guide with predetermined question areas such as “Can you describe your work environment at SHD?” The participant was also asked about “What obstacles do you experience in your work environment” and “What prerequisites are important in your work environment”. Probing questions such as “Can you please tell me more?” were asked to deepen the telenurses’ descriptions of their reflections on their work environment [31]. The interviews were conducted from March until May 2015; 15 interviews were conducted via personal meeting and 9 interviews were conducted via telephone by the first author. The interviews lasted between 33 and 107 min (mean time 62.7 min) and were recorded on an MP3 player. After the qualitative data analysis, the identified categories (henceforth *items)*, indicating obstacles and prerequisites were sent to the participants and an additional 106 telenurses using a web survey (Sun-net Survey) for ranking based on importance on a 7-grade Likert scale (Round II), (1 = not at all important, 2 = low importance, 3 = slightly important, 4 = moderately important, 5 = important, 6 = very important, 7 = extremely important). Criterion for consensus and re-rating in Round III was set to at least 55% of the participating telenurses rating an item as 5 (important) to 7 (extremely important). Items (obstacles and prerequisites) showing consensus (>55%) were selected for Round III and sent back to the participants. The results from Round II were, as recommended by Keeney et al. [29], then returned to the participants in the web survey, showing the overall group response from Round II. The participants were asked to reconsider their initial response from the previous round (Round II) and again rank the items (obstacles and prerequisites) based on their importance using the 7-grade Likert scale. For a description of the identified obstacles and important prerequisites and the ranking in Round II and III, see Tables 1 and 2. In Round II, 69 out of 130 (53%) participants completed the web survey, and in Round III 54 out of 69 (78.3%) did so.

**Table 1:** Important obstacles and prerequisites in telenurses’ work environment according to the findings in Round 1 (the interviews) presented alphabetically order

	Items of Obstacles	Example of content The participants described:
1	All of the working hours monitored	…all of their working hours were monitored and evaluated regarding number of answered calls, number of referrals and how long they had been logged off their work station.
2	Cognitive fatigue	…cognitive fatigue, the long working periods with the high level of concentration their work involved exhausted them. 8-h shift was perceived as far too long, and for this reason several of the participants had chosen to work part time.
3	Collaboration with other sites	…collaboration with other sites (regions) within SHD increased call length.
4	Disturbing sounds	…disturbing sounds such as loud voices disrupted concentration, taking attention away from the caller.
5	High employee turnover	…high employee turnover and subsequent shortage of staff were perceived as an obstacle because it was difficult to recruit new colleagues.
6	Lack of possibilities for development (professionally)	…lack of possibilities for development and opportunities for continuing education were perceived as low.
7	Lack of possibilities to discuss and reflect with colleagues	…lack of possibilities to discuss and reflect with colleagues
8	Lack of support and appreciation from managers	…lack of support and appreciation from managers, especially when something went wrong, e.g. when they received complaints from other healthcare providers or callers.
9	Lack of understanding and being exposed to critic from other healthcare providers	…lack of understanding and becoming exposed to critic from other healthcare providers regarding their assessments of callers’ care needs
10	Lacking availability among other healthcare providers	…lack of availability to other healthcare providers and they often encountered callers who were frustrated over other healthcare providers’ lack of availability.
11	Lacking availability to the callers’ medical charts	…they lacked availability to the callers’ medical charts and hence important medical information
12	Limited career opportunities	…limited career opportunities within their workplace.
13	Limited possibilities to socialize with colleagues,	…limited possibilities to socialize with colleagues, lunch and coffee breaks were almost always taken alone and opportunities for spontaneous socialization with colleagues were few.
14	Long telephone queues	…how long telephone queues were experienced as stressful and made them try to shorten calls to decrease waiting time, but this strategy increased their sense of insecurity.
15	Low possibility to affect work situation	…low possibility to affect their work situation, calls were automatically forwarded to them
16	Management did not avail themselves of the knowledge and experience	…management did not avail themselves of the knowledge and experience of employed telenurses
17	No feedback on one’s own calls	…feedback on one’s own calls was inadequate and scarce.
18	No occupational health services	…some did not have occupational health services and reported having no one to turn to regarding work stressors and associated problems
19	No opportunity for recovery during the work shift	…no opportunities for recovery during the work shift, it was almost impossible to just sit back for some minutes and rest when all of their working hours were being monitored.
20	Periodical repetitively work	…in some respects, the work was periodical repetitively, especially in periods of outbreaks of, e.g., influenza and gastroenteritis.
21	Problems with availability to an interpreter	…problems with availability of interpreter occurred as callers to SHD sometimes not are native speakers of Swedish.
22	Sedentary work cause physical strain	…their sedentary work caused physical strain and several experienced neck and shoulder pain
23	Solitary work	…how being a telephone advice nurse was solitary work; they worked alone at their computer using headsets without interacting with colleagues.
24	Technical problems	…technical problems with telephone equipment, computers and software aggravated the participants’ work.
25	The CDSS	*…the CDSS* was described as undesirable; it contained so much information that it was difficult to find specific information. Searching took time and attention away from the callers.
26	Unclear guidelines and lack of structure at the workplace	…they perceived problems with unclear guidelines and lack of structure at the workplace.
27	Working within a production-steered business with demands for efficiency	…they perceived their work environments to be like working within a production-steered business with demands for efficiency.
28	Wrong kind of calls	… they sometimes had to handle the wrong kind of calls, e.g., calls regarding opening hours at pharmacies, administrative questions regarding medical bills, etcetera
	Items of Prerequisites	Example of content The participants described how:
29	All calls to SHD were recorded and that the content of each call was clearly documented	…the fact that all calls to SHD were recorded and that the content of each call was clearly documented was described as valuable and as contributing to having a sense of control.
30	Being able to call callers back	…being able to call callers back to follow up on their condition. This increased participants’ feelings of control and reduced their anxiety about making incorrect assessments.
31	Being able to choose the size of their workroom	…being able to choose the size of their workroom, e.g. decide whether they wanted to sit alone or with colleagues was desired
32	Being able to focus on one caller at a time	…having one patient at a time gave a feeling of control, whereas in traditional care they were supposed to manage several patients at the same time.
33	Calm and pleasant environment	…working in an environment without disturbing sounds and pleasant surroundings .
34	Collegial support	…a permissive atmosphere with possibilities for social interactions with colleagues enabled collegial support, which was described as essential to their work, especially after handling difficult calls.
35	Education and observation possibility	…having possibility to education and observation strengthened telenurses and developed their professional skills.
36	Health and wellness training/massage	… was appreciated and eased the physical strain caused by their work.
37	Joint coffee and lunch breaks	…joint coffee and lunch breaks provided an important chance for social interaction.
38	Possibility to adjust the workstation	…possibility to adjust the workstation e.g., to stand up and work, was regarded as important in avoiding/preventing physical strain.
39	Possibility to influence work time	…those given the possibility to influence their work time felt this was important since it enabled them to work when it suited their private and family life.
40	Regular feedback on one’s own calls	…regular feedback on one’s own calls was a prerequisite for professional development. Listening to one’s own calls with a coach allowed them to observe and reflect on their communication with the callers and develop their communicative skills.
41	Stimulating work	…they perceived a stimulating work, every call was an unexpected challenge and every caller was unique. The callers often expressed their appreciation, and the participants described how they felt they could help the callers in important ways.
42	Structure and support from the “Call dialogue	…the *“Call dialogue*” helped to focus on the callers’ problem and ensure that nothing important had been left out
43	Structure and support via CDSS	…the CDSS gave participants access to structure and support, hence as a tool to structure the call and support their assessments through the system.
44	Support from management	…support from management was an important prerequisite and created a feeling of security, as they knew they always had someone to turn to, especially when they were exposed to criticism from other healthcare providers.
45	Technical support 24/7	…adequate and professional technical support 24/7

**Table 2:** Important obstacles and prerequisites in telenurses’ work environment according to, and graded by telenurses in round II and III

No	Round I	Round II				Round III			
	Items of Obstacles	Md	Q1-Q3	Mean	SD	Md	Q1-Q3	Mean	SD
1	All of the working hours monitored	4.0	3.0;5.0	4.1	2.0	-	-	-	-
2	Cognitive fatigue	6.0	5.0;7.0	5.8	1.6	6.0	5.0;7.0	5.8	1.5
3	Collaboration with other sites	3.0	1.0;5.0	3.3	2.1	-	-	-	-
4	Disturbing sounds	6.0	3.5;7.0	5.1	2.0	5.0	5.0;7.0	5.4	1.8
5	High employee turnover	5.0	4.0;7.0	5.0	1.8	5.0	3.0;6.0	4.6	1.8
6	Lack of possibilities for development (professionally)	5.0	4.0;7.0	5.0	1.9	4.5	3.0;6.0	4.5	1.7
7	Lack of possibilities to discuss and reflect with colleagues	5.0	3.0;6.0	4.7	1.8	5.0	4.0;6.0	5.0	1.5
8	Lack of support and appreciation from managers	6.0	5.0;7.0	5.3	1.8	5.0	4.0;7.0	5.2	1.6
9	Lack of understanding and being exposed to critic from other healthcare providers	5.0	4.0;6.0	4.9	1.7	5.0	4.0;6.0	4.6	1.8
10	Lacking availability among other healthcare providers	6.0	4.0;7.0	5.3	1.6	6.0	4.5;7.0	5.4	1.7
11	Lacking availability to the callers’ medical charts	2.0	1.0;3.0	2.4	1.7	-	-	-	-
12	Limited career opportunities	4.0	2.5;6.0	4.2	2.0	-	-	-	-
13	Limited possibilities to socialize with colleagues,	5.0	4.0;6.0	4.6	1.7	5.0	3.0;6.0	4.5	1.8
14	Long telephone queues	5.0	3.0;6.0	4.7	1.9	5.0	3.5;6.0	4.6	1.8
15	Low possibility to affect work situation	5.0	3.0;6.0	4.8	1.8	5.0	4.0;6.5	5.0	1.7
16	Management did not avail themselves of the knowledge and experience	4.0	3.0;5.5	4.2	1.8	-	-	-	-
17	No feedback on one’s own calls	5.0	3.0;6.0	4.8	1.8	5.0	3.5;6.0	4.8	1.9
18	No occupational health services	3.0	1.5;4.0	3.2	1.8	-	-	-	-
19	No opportunity for recovery during the work shift	6.0	4.0;7.0	5.3	1.9	6.0	5.0;7.0	5.4	1.5
20	Periodical repetitively work	5.0	3.0;6.0	4.6	1.8	5.0	4.0;6.0	4.8	1.6
21	Problems with availability to an interpreter	3.0	2.0;5.0	3.4	2.0	-	-	-	-
22	Sedentary work cause physical strain	5.0	4.0;7.0	5.1	1.8	6.0	5.0;7.0	5.3	1.8
23	Solitary work	3.0	1.0;4.0	3.0	1.9	-	-	-	-
24	Technical problems	6.0	3.5;7.0	5.1	1.9	6.0	5.0:7.0	5.5	1.5
25	The CDSS	4.0	1.5;6.0	3.9	2.0	-	-	-	-
26	Unclear guidelines and lack of structure at the workplace	6.0	4.0;7.0	5.2	1.8	4.5	3.0;6.0	4.4	1.7
27	Working within a production-steered business with demands for efficiency	5.0	3.0;6.0	4.5	1.8	5.0	4.0;6.0	4.8	1.6
28	Wrong kind of calls	4.0	2.0;6.0	3.7	2.1	-	-	-	-
	Items of prerequisites	Md	Q1-Q3	Mean	SD	Md	Q1-Q3	Mean	SD
29	All calls to SHD were recorded and that the content of each call was clearly documented	6.5	6.0;7.0	6.0	1.5	6.0	5.0;7.0	5.6	1.5
30	Being able to call callers back	5.0	4.0;7.0	5.2	1.8	5.0	3.0;6.0	4.6	1.9
31	Being able to choose the size of their workroom	6.0	5.0;7.0	5.6	1.9	7.0	5.0;7.0	5.9	1.6
32	Being able to focus on one caller at a time	7.0	6.5;7.0	6.7	0.7	7.0	6.0;7.0	6.5	0.9
33	Calm and pleasant environment	7.0	6.0;7.0	6.4	1.0	7.0	6.0;7.0	6.4	1.0
34	Collegial support	7.0	5.0;7.0	6.0	1.2	6.0	6.0;7.0	5.9	1.3
35	Education and observation possibility	6.0	5.5;7.0	6.1	1.2	6.0	5.0;7.0	5.7	1.4
36	Health and wellness training/massage	6.0	4.0;7.0	5.3	1.8	6.0	5.0;7.0	5.3	1.8
37	Joint coffee and lunch breaks	5.0	4.0;6.0	4.9	1.6	5.0	3.0;6.0	4.6	1.8
38	Possibility to adjust the workstation	7.0	6.0;7.0	6.2	1.3	6.0	6.0;7.0	6.2	0.9
39	Possibility to influence work time	7.0	6.0;7.0	6.2	1.3	7.0	6.0;7.0	6.3	1.0
40	Regular feedback on one’s own calls	5.0	4.0;7.0	5.1	1.7	6.0	4.0:6.0	5.5	1.5
41	Stimulating work	7.0	6.0;7.0	6.4	1.0	6.0	6.0;7.0	6.2	1.2
42	Structure and support from the “Call dialogue	6.0	5.0;7.0	5.5	1.6	6.0	4.0;7.0	5.4	1.6
43	Structure and support via CDSS	7.0	6.0;7.0	6.2	1.2	4.0	1.5;6.0	6.1	1.3
44	Support from management	7.0	6.0;7.0	6.4	0.9	6.0	5.0;7.0	5.9	1.3
45	Technical support 24/7	7.0	6.0;7.0	6.3	1.2	7.0	6.0;7.0	6.2	1.2

“-” = not included in Round II based on rating in Round II

Presented ad median values, quartiles, mean values and standard deviations comparing the second and third round questionnaires

### Data analysis

The interview data were analysed by the first and last author using manifest qualitative content analysis [32]. All interviews were transcribed verbatim, read and re-read several times to get a sense of the content. The interview text were put into a single text word-document and analysed using an inductive qualitative content analysis in line with Elo and Kyngäs [33]. Words and sentences e.g. meaning units related to obstacles and prerequisites were extracted, condensed and abstracted and labelled with a code that described the content of the meaning unit. The codes were sorted into categories based on similarity and differences and 45 categories (henceforth *items)* were found. Categories was formulated with the ambition of remaining close to the concepts and words used in the respondents’ answers. Theses 45 items consisted of 28 items of obstacles and 17 items of prerequisites, see Table 1. To achieve acceptable methodological quality the analysis was an on-going process involving all authors. Quantitative data from the web survey were entered into IBM SPSS Statistics for Windows for analysis, and descriptive statistics were used to explore median (Md), mean and quartiles (Q) as well as frequencies and proportions for individual items and ranking. Criterion for consensus and re-rating in Round III was set to at least 55% of the participating telenurses rating an item as 5 (important) to 7 (extremely important) [29]. All data collection was performed by the same author, using an interview-guide to ensure that all participants were asked the same questions. To enhance dependability all of the interviews were made by one author (AE). Data analysis was performed by two authors (AE and ACW) in order to strengthen credibility of the findings. Discussions were held until consensus was reached regarding names for the items. The result of the analysis, hence the identified items were presented to telenurses and managers within SHD for establishing face-validity on two occasions.

## Results

Data collection and the results from each round of the Delphi are presented in the results section owing to the iterative nature of the study. The results are based on the interviews (Round I) and the result of the responses collected in the two questionaries’ (Round II and III).

### Round I

The initial interviews were conducted with 24 telenurses, all employed within SHD. Analysis of the qualitative interview data resulted in a total of 45 items, 28 items of obstacles and 17 items of prerequisites, see Table 1. This study finds a variety of important obstacles and prerequisites within telenurses work environment. These 45 items are a description of the important obstacles and prerequisites the telenurses expressed within the interviews. To enable consensus on important obstacles and prerequisites in telenurses work environment the results, 45 items of obstacles and prerequisites, identified in the interviews were entered into a web-survey (Sun-net Survey) and sent back to the 24 telenurses participating in the interviews and additional 106 telenurses for ranking based on importance on a 7-grade Likert scale (Round II).

### Round II

A total 69 (out of 130 (53%)) participants completed the web survey in Round II. As one of the aims of the study was to achieve consensus on the important factors in the work environment, the authors agreed to set the criterion for consensus in Round II, and thereby re-rating in Round III, to at least 55% of the participating telenurses rating an item as 5 (important) to 7 (extremely important) [30]. In Round II, 35 out of 45 items met the pre-determined acceptance for Round III criterion. The median values of respondents’ ratings of the 28 items in Round II regarding important Obstacles varied from “*not having the callers’ medical charts*”(nr.11) (Md = 2.0) to the seven items “*cognitive fatigue*”(nr.2), “*unclear guidelines and lack of structure at the workplace*”(nr.26), “*no opportunity for recovery during the work shift*”(nr.19), “*lacking availability among other healthcare providers*”(nr.10), “*lack of support and appreciation from managers*”(nr.8), “*disturbing sounds*”(nr.4) and “*technical problems*”(nr.24) (Md = 6.0). The median values for important Prerequisites varied from “*regular feedback on one’s own calls*”(nr. 40) and “*being able to call callers back*” (nr.30)(Md = 5.0) to the nine items “*being able to focus on one caller at a time”(nr.32), “possibility to influence their work time“(nr.39), “structure and support* via *CDSS”(nr. 43), “support from management”(nr.44), “calm and pleasant environment”(nr.33), “collegial support”(nr.34),”having a stimulating work”(nr. 41),”possibility to adjust the work station*” (nr.38)and “*technical support*” (nr.45)(Md = 7.0); see Table 2.

### Round III

In Round III, the web survey was structured with the highest ranked item placed first together with the ranking the item received in Round II. The analysis of the final round resulted in an interdependent ranking of the 18 items regarding important Obstacles, which varied from “*unclear guidelines and lack of structure at the workplace*” (no. 26) (Md = 4.5) to “*cognitive fatigue*” (no.2), “*no opportunity for recovery during the work shift*” (no.19) and “*lacking availability among other healthcare providers*” (no.10) (Md = 6.0); see Table 1. The median values for important Prerequisites varied from “*joint coffee and lunch breaks*” (no.37) and “*being able to call callers back*” (no.30) (Md = 5.0) to the five items “*ability to focus on one caller at a time”(*no.32*),” possibility to influence work time”(*no.39*),” being able to choose the size of their workroom”(no.31), “calm and pleasant environment*”(no.33) and “*technical support 24/7*”(no.45) (Md = 7.0); see Table 2. The items, e.g. obstacles (11 items) and prerequisites (10 items) receiving the highest rank in Round III are presented in Tables 3 and 4. Two items (item 10 and item 11) of obstacles received the same ranking and thereby 11 items for obstacles.

**Table 3:** Eleven most important obstacles in telenurses’ work environment as found in round III

	Obstacles	Md	Q1-Q3	Mean	SD
1	Cognitive fatigue	6.0	5.0;7.0	5.8	1.5
2	Technical problems	6.0	5.0;7.0	5.5	1.5
3	No opportunity for recovery during the work shift	6.0	5.0;7.0	5.4	1.5
4	Lacking availability among other healthcare providers	6.0	4.5;7.0	5.4	1.7
5	Sedentary work cause physical strain	6.0	5.0;7.0	5.3	1.8
6	Disturbing sounds	5.0	5.0;7.0	5.4	1.8
7	Lack of support and appreciation from managers	5.0	4.0;7.0	5.2	1.6
8	Lack of possibilities to discuss and reflect with colleagues	5.0	4.0;6.0	5.0	1.5
9	Periodical repetitively work	5.0	4.0;6.0	4.8	1.6
10	Working within a production-steered business with demands for efficiency	5.0	4.0;6.0	4.8	1.6
11	Low possibility to affect work situation	5.0	3.0;6.0	5.0	1.7

**Table 4:** Ten most important prerequisites in telenurses’ work environment as found in round III

	Prerequisites	Md	Q1-Q3	Mean	SD
Being able to focus on one caller at a time	7.0	6.0;7.0	6.5	0.9	0.9
Calm and pleasant environment	7.0	6.0;7.0	6.4	1.0	1.0
Possibility to influence work time	7.0	6.0;7.0	6.3	1.0	1.0
Having technical support 24/7	7.0	6.0;7.0	6.2	1.2	1.2
Possibility to adjust the workstation	7.0	6.0;7.0	6.2	0.9	0.9
Stimulating work	6.0	6.0;7.0	6.2	1.2	1.2
Structure and support via CDSS	4.0	6.0;7.0	6.1	1.3	1.3
Being able to choose the size of their workroom	7.0	5.0;7.0	5.9	1.6	1.6
Support from management	6.0	5.0;7.0	5.9	1.3	1.3
Collegial support	6.0	6.0;7.0	5.9	1.3	1.3

## Discussion

The findings from the current Delphi study present a consensus view of telenurses’ experiences of obstacles and prerequisites in their work environment. Central to the findings are the aspects of “*cognitive fatigue*”(no.2), “*having no opportunity for recovery during the work shift*”(no.19) and “*lacking availability among other healthcare providers*” (no.10) all received the highest rankings in Round II as well as Round III. In a previous study [18], the results showed that the main reason for medical errors within SHD was telenurses failing to listen to the caller and asking too few questions, perhaps as a result of cognitive failure and tiredness. According to the telenurses, stress and fatigue were involved in the malpractice claims and were described as contributing factors [34]. The identified problem with lacking availability among other healthcare providers has been described in a previous study [23], where it was the most common reason for an incident report within SHD.

Three of the highest ranked items regarding prerequisites in both Round II and III were related to having control of the work situation, hence the “*being able to focus on one caller at a time*”(no.32), “*possibility to influence work time*”(no.39) and “*being able to choose the size of their workroom*”(no.31). Having a sense of control is an important factor for healthy work [16]. Many of the items receiving high ranks in Round II and III regard lack of control of the work situation, and based on evidence in the literature, they should be addressed by the organizations employing the telenurses to prevent work-related illness. The control and demand model [16] explains that the effects of psychological demands co-vary with the degree of control, e.g. decision latitude. The effect of psychological demands also co-varies with the level of support offered to employees by the organization. According to the model [16], the worst work situation is when employees have high demands and low levels of support combined with low possibility to influence and affect their work situation, e.g., iso-strain. Today, there is consensus in the research that iso-strained work is a risk factor for development of heart disease and psychiatric illness. Telenurses are subjected to many conflicting demands in their work [7], and it is necessary to increase their ability to control their work, because high demands can be manageable when combined with control [16]. The results of the present study could also be reflected on the findings of the Job Demand – resources model (JD-R) that suggests that working conditions can be categorized into two different categories: job demands and job resources [35]. The obstacles and prerequisites found within the present study could be regarded as examples of these kind of job demands and job resources. Job demands includes the aspects of work that requires sustained cognitive and emotional efforts from the employee and these demands are associated with costs for the individual [36]. These kind of costs were explicit found within the present study whereas *“cognitive fatigue*” (no.2) and “*having no opportunity for recovery during the work shift*” (no.19) was two of the highest ranked items in Round III.

The present results also revealed high levels on items related to lack of support, from managers as well as from other healthcare providers. Support is an important part of Karasek and Theorell’s model [16]; they state that low levels of support, excessive demands and poor decision latitude lead to mental fatigue as well as low back pain. In the present study, the participants indicated that sedentary work, which leads to physical strain, is an obstacle in their work. But based on Karasek and Thorell’s model [16], other factors – e.g., lack of support and appreciation from managers (no.8), lack of understanding and being exposed to critic from other healthcare providers (no.9) – may also cause their experience of physical strain, not only the fact that their work is sedentary. Having support was ranked as important; both collegial support (no.34) and support from management (no. 44) were ranked as important items in Round II and III. A previous study showed how collegial support affected job performance and could also enhance the quality of care. The study also showed that nurses with high social support had lower perceived job stress and increased nurse retention [37]. The most common sources of stress in nursing care include a high workload, poor collegial support, role conflict and role ambiguity. These factors are similar for all nurses irrespective of type of ward or nursing specialty and would appear to be inherent to the nursing role [38]. In the present study, three of these factors were described, e.g., high workload, poor collegial support and ambiguity on how to preform work (e.g. unclear guidelines). Coaching and task support are the types of social support most predictive of job tenure [38]. In the present study, these were regarded as important prerequisites and as obstacles when they were not available.

### Limitations

One strength of the present study is the prolonged engagement of the participants throughout the course of the study. As there was no previous research on the topic, an exploratory approach using Delphi technique was chosen [29, 39]. The technique has been shown to be flexible and to have several advantages when searching for consensus on a specific topic. Because there are no recommendations regarding the appropriate sample sizes for the different phases, a decision was made to include 24 experts (telenurses) in the first qualitative round. In Round I, all participating telenurses were female, which may limit the generalizability of the study. Male telenurses were included in Round II and III, which could be regarded as a strength. Due to the small sample, it was not possible to study differences between groups based on gender and age. The stability of the data, e.g. the small changes in item ratings in Round II and III, indicates that the identified items are representative of the context. The response rate for Round II was limited (53.1%). However, the 106 new telenurses had not been asked about their interest in participating in the study prior to sending out the web survey. But regarding the respective groups, of the 24 telenurses participating in Round I, 21 completed Round II. In Round III, the overall response rate was 54 out of 69 (78.3%). This indicates the importance of including participants early in the study, because this decreases dropout. The level for consensus in Round II was set at 55% of participants ranking the item 5–7 in a 7-grade Likert scale. The literature provides few clear guidelines on what consensus to set. Depending on the importance of the topic, the literature suggests consensus levels varying from 100% in life-and-death issues to Loughlin and Moore’s [39] suggestion that consensus be equated with 51% agreement among respondents. In Round III, we decreased the number of items and only included items that were ranked as important by more than 55% of participants in Round II. The reason for the reduction was to avoid duplication and to reduce participant fatigue [40]. For establishing face-validity the result of the analysis, hence the identified items were presented to telenurses and managers within SHD at two occasions.

## Conclusions

Managers need to enable telenurses to experience control in their work. Given the nature of telenursing, e.g., high levels of demand and subsequent tiredness due to long working periods, telenurses need to be provided with possibilities to control their work and to recover during work. Moreover, shortening work time could improve their work environment. Healthcare organizations (politicians) need to address the problems associated with limited resources, because lacking availability among other healthcare providers has long been a problem in Swedish Healthcare. Limited possibilities to perform work might contribute to feelings of stress and inability to perform work.
